# Learner-centered education: ICU residents’ expectations of teaching style and supervision level

**DOI:** 10.1186/s12909-021-02844-z

**Published:** 2021-07-31

**Authors:** Bjoern Zante, Jennifer M. Klasen

**Affiliations:** 1 Department of Intensive Care Medicine, Inselspital, Bern University Hospital, University of Bern, Freiburgstrasse 10, 3010 Bern, Switzerland; 2 Clarunis, Department of Visceral Surgery, University Centre for Gastrointestinal and Liver Diseases, University Hospital Basel, Basel, Switzerland

**Keywords:** Education, Critical care, Technical skills, Procedural skills, Entrustable professional activities, EPA

## Abstract

**Background:**

If the education of intensive care unit (ICU) residents focuses on individual learning behavior, the faculty’s style of teaching and level of supervision need to be adapted accordingly. The aim of this study was to delineate the associations between residents’ perceived learning behavior, experience, and demographics and their expectations with regard to teaching style and supervision levels.

**Methods:**

This multicenter survey obtained data on ICU residents’ base specialty, duration of ICU training, individual postgraduate year, gender, and number of repetitions of ICU skills. Using 4-point Likert scales, residents assessed perceived learning behavior, expected teaching style, and supervision level for respective skills. Multivariate regression analysis was used to evaluate associations between assessed variables.

**Results:**

Among 109 residents of four interdisciplinary ICUs, 63 (58%) participated in the survey and 95% (60/63) questionnaires were completed. The residents’ perceived learning behavior was associated with number of skill repetitions (*p* < 0.0001), internal medicine as base specialty (*p* = 0.02), and skill type (*p* < 0.0001). Their expected teaching style was associated with learning behavior (*p* < 0.0001) and skill type (*p* < 0.0001). Their expected supervision level was associated with skill repetitions (*p* < 0.0001) and skill type (*p* < 0.0001).

**Conclusion:**

For effective learner-centered education, it appears useful to recognize how the residents’ learning behavior is affected by the number of skill repetitions and the skill type. Hence, faculty may wish to take into account the residents’ learning behavior, driven mainly by skill complexity and the number of skill repetitions, to deliver the appropriate teaching style and supervision level.

**Supplementary Information:**

The online version contains supplementary material available at 10.1186/s12909-021-02844-z.

## Introduction

Residents in intensive care medicine have to acquire competency in various skills with the aid of training programs [1]. Earlier styles of teaching, such as William Halsted’s “see one, do one, teach one” [2] have been replaced over time by more theory-driven concepts. These theory-driven concepts (operant learning, neuropsychological theory of motor skill learning, cognitivism) differ in the emphasis they place on the roles of the faculty and the learners in the process of teaching and/or learning [3–6]. Current ICU teaching concepts have so far neglected the association of faculty’s and learners’ behavior [1, 7, 8].

Hersey et al. developed the Situational Leadership Theory to achieve effective leadership behavior in management organizations [9]. In brief, it is defined as “interplay among (1) the amount of guidance and direction a leader gives; (2) the amount of socioemotional support relationship behavior a leader provides; and (3) the readiness that individuals exhibit in performing a specific task [10]. This model suggests that the style of leadership should be adapted according to the followers’ behavior. It seems natural to adapt this model to the medical education setting, since even teaching requires significant leadership competency [11] and current teaching concepts have so far neglected the association of faculty’s and learners’ behavior [12, 13].

**Fig. 1:**
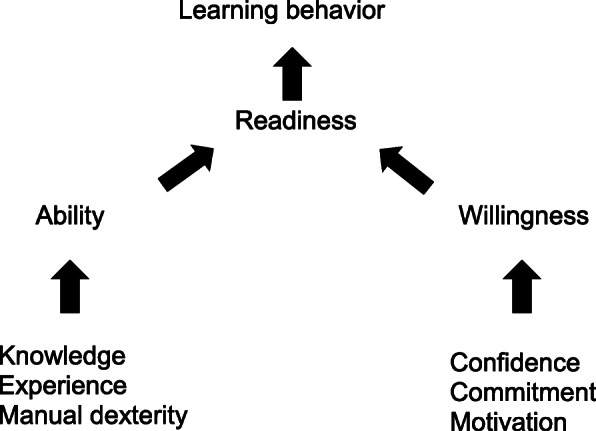
Conditions of ability and willingness drive learning behavior (adapted from Hersey et al.’s *Situational Leadership Theory* [14]

Therefore, instead of using only one teaching style faculty should be able to adapt their methods depending on the learners’ (residents’) learning behavior. Differences in learning behavior are dependent on the learners’ readiness (combination of ability and willingness, Fig. 1) and various teaching styles may be appropriate: (1) directing style, (2) coaching style, (3) supporting style, and (4) delegating style (corresponding learning behaviors and teaching styles are given in Table 1) [14]. It appears the readiness and therefore the distinct learning behavior may develop over time and therefore it would seem appropriate to adjust the teaching style accordingly [14]. In conventional class lecturing teaching styles may shift from “directing” (teacher in front of the class), through “coaching” (teacher directing the conversation), to “support” (teacher participating as a supportive nondirective group member), and in the final “delegating” style the teacher is only involved when asked by the group [14]. However, the adaptation of this concept to individual teaching of procedural ICU-skills remains unclear.

**Table 1:** Corresponding learning behaviors and teaching styles

**Learning behavior**	**Teaching style**
**Novice**	**Directing style**
- Not performing task to acceptable level - Being intimidated by task - Being unclear - Procrastination - Asking questions about task - Avoiding task or frustration - Being defensive or uncomfortable	- Providing specifics: who, what, when, where and how - Close supervision and accountability - Incremental instructions - Keep it simple and specific - Guiding, telling, directing - Predominantly one-way communication
**Advanced Beginner**	**Coaching style**
- Anxious or excited - Interested and responsive - Demonstrating moderate ability - Receptive to input - Attentive - Enthusiastic - New task, no experience	- Providing specifics: who, what, when, where, how and why - Need for explaining decisions, and clarification - Ask questions to clarify ability level - Reinforce small improvements - Two-way dialogue - Explaining, clarifying, persuading
**Competent**	**Supporting style**
- Demonstrated knowledge and ability - Appears hesitant to finish or take next step - Seems reluctant to perform alone - Solicits frequent feedback	- Encourage input - Actively listening - Two-way communication and involvement - Support risk-taking - Compliment work - Praise and build confidence - Participating, encouraging, supporting, empowering
**Proficient**	**Delegating style**
- Keeps teacher informed of task progress - Can operate autonomously - Is result-orientated - Shares both good and bad new - Make effective decisions regarding task - Performs to high standards - Is aware of expertise	- Delegating task - Follower-made decisions - Relatively light supervision - Monitor activities - Reinforce results - Remain accessible - Delegating, observing, entrusting, assigning

Corresponding sets of learning behaviors and teaching styles were adapted from Hersey et al.’s *Situational Leadership Theory* [14]

Besides the use of appropriate teaching styles, learner-centered education demands adaptation of the level of supervision to the prevailing circumstances. Here, Ten Cates’ entrustable professional activities (EPA) combine formative assessments to enhance the learners’ performance and summative assessments to define the appropriate supervision level depending on the learners’ competency [15, 16]. Based on individuals’ competencies, different supervision levels may apply: (1) observation but not execution, even with direct supervision; (2) execution with direct, proactive supervision; (3) execution with reactive supervision, i.e., on request and quickly available; (4) supervision at a distance and/or post hoc [15]. In several EPA concepts, however, it remains elusive how one can move up to the next supervision level [17, 18]. Even in other concepts for skill training, the summative assessment of competencies remains unclear [19, 20].

In this multicenter study, we set out to explore associations between intensive care unit (ICU) residents’ perceived learning behavior, experience, and demographics and their expectations with regard to teaching style and supervision levels regarding typical ICU procedural skills.

## Material and methods

### Participants

The participants in this cross-sectional multicenter survey were residents working in multidisciplinary departments of intensive care medicine at four tertiary care teaching hospitals. The residents’ base specialties were internal medicine, surgery, anesthesiology, intensive care medicine, and others. Their duration of training in intensive care medicine varied from 6 months or less to more than 12 months. The survey was administered by means of an online tool (UmfrageOnline, enuvo GmbH, Zuerich, Switzerland). The survey was launched in April 2020, followed by two reminders at intervals of 4 weeks. Participation was voluntary and anonymous. The Ethics Committee of Bern waived the need for ethics approval and the need to obtain consent for the collection, analysis and publication of the data for this study (Req-2020-01350). This investigation adhered to the tenets of the Declaration of Helsinki.

### Assessment of residents’ expectations

The survey (given as supplementary file) recorded the following demographic indices: gender, postgraduate year (PGY), base specialty, and duration of ICU training. Furthermore, the residents were asked about their individual experience in typical ICU skills (chest drain insertion, tracheostomy, cricothyroidotomy, pericardiocentesis, insertion of ECMO cannulas, endotracheal intubation, central venous line insertion, and arterial line insertion) stated as number of skill repetitions performed. The number of repetitions for each skill was classified as follows: zero repetitions, 1–5 repetitions, 6–10 repetitions, 11–20 repetitions, 21–50 repetitions, and more than 50 repetitions.

Residents were surveyed regarding their self-perceived learning behavior (Table 1) and their expectations of faculty teaching style regarding the different skills (Table 1). The wording and the combinations of the different behaviors and the corresponding expected/needed teaching/leading styles are taken directly from the original Situational Leadership Theory by Hersey et al. [14]. They were also asked about their expectations concerning EPA supervision levels for the different skills: (1) observation but not execution, even with direct supervision; (2) execution with direct, proactive supervision; (3) execution with reactive supervision, i.e., on request and quickly available; (4) supervision at a distance and/or post hoc [15].

### Statistical analysis

Statistical analysis was performed using STATA 16.1 (StataCorp. 2019. *Stata Statistical Software: Release 16*. College Station, TX: StataCorp LLC.).

Univariable and multivariable mixed-effects linear models were run with the respective outcome (learning behavior, teaching style and supervision level) as dependent variable and gender, PGY, duration of training, medical base specialty, skill type, and skill repetitions as independent variables. In the models for teaching style and supervision level, learning behavior was additionally used as an independent variable. Random terms for hospitals and participants were introduced to the models to account for correlation of data within hospitals and within participants. We fitted models via maximum likelihood and used an independent variance-covariance structure. Pairwise Spearman correlations between ordinal and binary covariates were below 0.52 indicating lack of collinearity. Residuals of multivariable models were inspected by means of Q-Q plots and residual-versus-fitted plots in order to detect outliers and deviations from normality. After removal of five outliers, normality assumptions were fulfilled. In order to account for multiple testing and control the family-wise type-I error rate at the nominal level of 0.05, we applied the Hochberg procedure to correct all *p*-values (*n* = 53) that were derived from the multivariable models [21]. The final dataset contained 480 observations, composed of the number of participating residents (*n* = 60) and the number of queried skills (*n* = 8).

## Results

Sixty three (58%) of the 109 residents participated in the survey, and 95% of the questionnaires (60/63) were completed. The three participants with missing outcome values were excluded from analysis.

### Demographics

Fifty percent (30/60) of participating residents were female (male: 50%, 30/60). The mean PGY was 6.61 years (standard deviation (SD) 3.38 years, range 2–22 years). The residents’ primary background was intensive care medicine (30%, 18/60), internal medicine (31.67%, 19/60), anesthesia (21.67%, 13/60), surgery (11.67%, 7/60), and other disciplines (5%, 3/60). The duration of ICU training was < 6 months for 40% (24/60), 7 to 12 months for 18% (11/60), and > 12 months for 42% (25/60) of the residents.

The residents’ characteristics were given according to perceived learning behavior (Table 2). The level of perceived learning behavior shifted from a lower to a higher level with (1) more postgraduate years; (2) the duration of training; (3) the number of skill repetitions. Residents with the base specialties intensive care medicine and anesthesiology rated perceived learning behavior as high, while residents of internal medicine rated perceived learning behavior as lower. With increasing skill complexity there was a shift from higher to lower level of perceived learning behavior.

**Table 2:** Demographics and characteristics according to residents’ perceived learning behavior

	Novice	Advanced Beginner	Competent	Proficient
Gender (female, %)	36 (50)	97 (51.87)	36 (43.9)	71 (51.07)
Postgraduate year	5.46 + 2.47	6.26 + 3.18	6.63 + 2.93	7.68 + 3.91
Duration of training				
- 1–6 months (%)	41 (56.94)	87 (46.52)	26 (31.71)	38 (27.34)
- 7–12 months (%)	11 (15.28)	38 (20.32)	15 (18.29)	24 (17.27)
- > 12 months (%)	20 (2.78)	62 (33.16)	41 (50)	77 (55.4)
Base specialty				
- Intensive care medicine (%)	12 (16.7)	50 (26.7)	27 (32.9)	55 (39.6)
- Anesthesiology (%)	8 (11.1)	34 (18.2)	21 (25.6)	41 (29.5)
- Internal medicine (%)	43 (59.7)	63 (33.7)	20 (24.4)	26 (18.7)
- Surgery (%)	4 (5.6)	29 (15.5)	10 (12.2)	13 (9.4)
- Others (%)	5 (6.9)	11 (5.9)	4 (4.9)	4 (2.9)
Skill repetitions				
- 0 (%)	71 (98.6)	146 (78.1)	16 (19.5)	3 (2.2)
- 1–5 (%)	1 (1.4)	38 (20.3)	41 (50.0)	16 (11.5)
- 6–10 (%)	0 (0)	2 (1.1)	15 (18.3)	17 (23.0)
- 11–20 (%)	0 (0)	0 (0)	8 (9.8)	32 (23.0)
- 21–50 (%)	0 (0)	0 (0)	2 (2.4)	24 (17.3)
- > 50 (%)	0 (0)	1 (0.5)	0 (0)	47 (33.8)
Skill type				
- Arterial line insertion (%)	0 (0)	2 (1.1)	6 (7.3)	52 (37.4)
- Central line insertion (%)	0 (0)	5 (2.7)	15 (18.3)	40 (28.8)
- Chest drain insertion (%)	2 (2.8)	25 (13.4)	16 (19.5)	17 (12.2)
- Endotracheal intubation (%)	5 (6.9)	17 (9.1)	12 (14.6)	26 (18.7)
- Tracheotomy (%)	10 (13.9)	31 (16.6)	15 (18.3)	4 (2.9)
- Cricothyroidotomy (%)	17 (23.6)	34 (18.2)	9 (11.0)	0 (0)
- ECMO cannula insertion (%)	19 (26.4)	34 (18.2)	7 (8.5)	0 (0)
- Pericardiocentesis (%)	19 (26.4)	39 (20.9)	2 (2.4)	0 (0)

The residents’ characteristics were given according to expected teaching style (Table 3). With increasing numbers of skill repetitions there was a shift from lower to higher levels of expected teaching style. With increasing skill complexity there was a shift from higher to lower level of expected teaching style.

**Table 3:** Demographics and characteristics according to residents’ expected teaching style

	Directing style	Coaching style	Supporting style	Delegating style
Gender (female, %)	81 (58.3)	47 (45.2)	44 (46.3)	68 (48.9)
Postgraduate year	5.62 + 2.12	6.69 + 4.02	6.55 + 2.59	7.58 + 4.0
Duration of training				
- 1–6 months (%)	69 (49.6)	57 (54.8)	26 (278.4)	38 (27.3)
- 7–12 months (%)	34 (24.5)	11 (10.6)	20 (21.1)	23 (16.5)
- > 12 months (%)	36 (25.9)	36 (34.6)	49 (51.6)	78 (56.1)
Base specialty				
- Intensive care medicine (%)	23 (16.5)	27 (26.0)	39 (41.1)	55 (39.6)
- Anesthesiology (%)	32 (23.0)	14 (13.5)	20 (21.1)	37 (26.6)
- Internal medicine (%)	68 (48.9)	34 (32.7)	21 (22.1)	29 (20.9)
- Surgery (%)	9 (6.5)	22 (21.2)	10 (10.5)	14 (10.1)
- Others (%)	7 (5.0)	7 (6.7)	5 (5.2)	4 (2.9)
Skill repetitions				
- 0 (%)	135 (97.1)	69 (66.3)	30 (31.6)	2 (1.4)
- 1–5 (%)	4 (2.9)	33 (31.7)	41 (43.2)	18 (12.9)
- 6–10 (%)	0 (0)	2 (1.9)	13 (13.7)	18 (12.9)
- 11–20 (%)	0 (0)	0 (0)	3 (3.2)	35 (25.2)
- 21–50 (%)	0 (0)	0 (0)	4 (4.2)	22 (15.8)
- > 50 (%)	0 (0)	0 (0)	4 (4.2)	44 (31.7)
Skill type				
- Arterial line insertion (%)	0 (0)	3 (2.9)	4 (4.2)	52 (37.4)
- Central line insertion (%)	0 (0)	5 (4.8)	13 (13.7)	42 (30.2)
- Chest drain insertion (%)	7 (5.0)	16 (15.4)	19 (20.0)	17 (12.2)
- Endotracheal intubation (%)	8 (5.8)	11 (10.6)	16 (16.8)	24 (30.2)
- Tracheotomy (%)	24 (17.3)	15 (14.4)	17 (17.9)	4 (2.9)
- Cricothyroidotomy (%)	32 (23.0)	16 (15.4)	12 (12.6)	0 (0)
- ECMO cannula insertion (%)	34 (24.5)	18 (17.3)	8 (8.4)	0 (0)
- Pericardiocentesis (%)	34 (24.5)	20 (19.2)	6 (6.3)	0 (0)

The residents’ characteristics were given according to expected supervision level (Table 4). With increasing numbers of skill repetitions there was a shift from lower to higher levels of expected supervision. With increasing skill complexity there was a shift from higher to lower level of expected supervision.

**Table 4:** Demographics and characteristics according to residents’ expected supervision level

	Novice	Advanced Beginner	Competent	Proficient
Gender (female, %)	90 (57.0)	62 (48.1)	34 (50.0)	53 (42.7)
Postgraduate year	5.67 ± 2.21	6.29 ± 2.8	6.74 ± 4.27	8.12 ± 3.99
Duration of training				
- 1–6 months (%)	83 (52.5)	54 (41.9)	24 (35.3)	30 (24.2)
- 7–12 months (%)	41 (25.9)	14 (10.9)	15 (22.1)	18 (14.5)
- > 12 months (%)	34 (21.5)	61 (47.3)	29 (42.6)	76 (61.3)
Base specialty				
- Intensive care medicine (%)	30 (19.0)	42 (32.6)	20 (29.4)	52 (41.9)
- Anesthesiology (%)	28 (17.7)	25 (19.4)	13 (19.1)	38 (30.6)
- Internal medicine (%)	71 (44.9)	39 (30.2)	21 (30.9)	20 (16.1)
- Surgery (%)	18 (11.4)	17 (13.2)	12 (17.6)	9 (7.3)
- Others (%)	11 (7.0)	6 (4.7)	2 (2.9)	5 (4.0)
Skill repetitions				
- 0 (%)	156 (98.7)	66 (51.2)	9 (13.2)	4 (3.2)
- 1–5 (%)	2 (1.3)	57 (44.2)	27 (39.7)	10 (8.1)
- 6–10 (%)	0 (0)	5 (3.9)	14 (20.6)	15 (12.1)
- 11–20 (%)	0 (0)	1 (0.8)	13 (19.1)	26 (21.0)
- 21–50 (%)	0 (0)	0 (0)	3 (4.4)	23 (18.5)
- > 50 (%)	0 (0)	0 (0)	2 (2.9)	46 (37.1)
Skill type				
- Arterial line insertion (%)	0 (0)	0 (0)	13 (19.1)	47 (37.9)
- Central line insertion (%)	0 (0)	7 (5.4)	17 (25.0)	36 (29.0)
- Chest drain insertion (%)	8 (5.1)	25 (19.4)	11 (16.2)	16 (12.9)
- Endotracheal intubation (%)	9 (5.7)	19 (14.7)	12 (17.6)	19 (15.3)
- Tracheotomy (%)	25 (15.8)	25 (19.4)	6 (8.8)	4 (3.2)
- Cricothyroidotomy (%)	34 (21.5)	21 (16.3)	3 (4.4)	2 (1.6)
- ECMO cannula insertion (%)	41 (25.9)	17 (13.2)	2 (2.9)	0 (0)
- Pericardiocentesis (%)	41 (25.9)	4 (5.9)	4 (5.9)	0 (0)

### Self-perceived learners’ behavior

Multivariable analysis indicated associations between self-perceived learning behavior and number of skill repetitions (*p* < 0.001), base specialty (*p* < 0.001), and skill type (tracheotomy, cricothyroidotomy, pericardiocentesis, and ECMO cannula insertion, all *p* < 0.001) (Table 5).

**Table 5:** Univariable models and multivariable models for dependencies of perceived learning behavior

Variable	Univariable models Crude coef. (95% CI)	***p***-value	Multivariable models Adjusted coef. (95% CI)	***p***-value	Corrected ***p***-value
Gender (female)	−0.01 (−0.27 to 0.24)	0.919	0.14 (− 0.03 to 0.32)	0.112	1.000
Postgraduate year	0.07 (0.04 to 0.11)	< 0.001	0.03 (− 0.00 to 0.05)	0.071	1.000
Duration of training	0.29 (0.16 to 0.41)	< 0.001	0.04 (−0.09 to 0.18)	0.521	1.000
Skill repetitions	0.50 (0.47 to 0.53)	< 0.001	0.29 (0.23 to 0.34)	< 0.001	**< 0.001**
Base specialty		< 0.001		< 0.001	**< 0.001**
- Intensive care medicine	Ref.		Ref.		
- Anesthesiology	0.01 (−0.25 to 0.27)	0.960	0.06 (− 0.17 to 0.29)	0.636	1.000
- Internal medicine	−0.78 (−1.03 to − 0.53)	< 0.001	−0.44 (− 0.69 to − 0.18)	0.001	**0.023**
- Surgery	− 0.32 (− 0.64 to − 0.00)	0.047	0.14 (− 0.18 to 0.46)	0.377	1.000
- Others	− 0.60 (− 1.05 to − 0.15)	0.009	−0.13 (− 0.58 to 0.32)	0.566	1.000
Skill type		< 0.001		< 0.001	**< 0.001**
- Chest drain insertion	Ref.		Ref.		
- Tracheotomy	−0.62 (− 0.81 to − 0.42)	< 0.001	− 0.50 (− 0.68 to − 0.32)	< 0.001	**< 0.001**
- Cricothyroidotomy	−0.93 (− 1.13 to − 0.74)	< 0.001	−0.63 (− 0.81 to − 0.45)	< 0.001	**< 0.001**
- Pericardiocentesis	−1.08 (− 1.28 to − 0.89)	< 0.001	−0.79 (− 0.97 to − 0.61)	< 0.001	**< 0.001**
- ECMO cannula insertion	−1.00 (− 1.20 to − 0.80)	< 0.001	−0.74 (− 0.92 to − 0.56)	< 0.001	**< 0.001**
- Endotracheal intubation	0.20 (0.01 to 0.40)	0.044	0.00 (− 0.18 to 0.18)	0.982	0.982
- Central line insertion	0.78 (0.59 to 0.98)	< 0.001	0.18 (−0.03 to 0.39)	0.094	1.000
- Arterial line insertion	1.03 (0.84 to 1.23)	< 0.001	0.32 (0.10 to 0.54)	0.004	0.128

The standard deviation of variance components of the multivariable model was 0.24, 0.23, and 0.49, respectively for hospitals, participants, and residuals

### Expected teaching style

Multivariable analysis indicated that teaching style was associated with perceived learning behavior (*p* < 0.0001) and skill type (tracheotomy, cricothyroidotomy, pericardiocentesis, and ECMO cannula insertion, all *p* < 0.001) (Table 6).

**Table 6:** Univariable models and multivariable models for dependencies of expected teaching style

Variable	Univariable models Crude coef. (95% CI)	***p***-value	Multivariable models Adjusted coef. (95% CI)	***p***-value	Corrected ***p***-value
Gender (female)	− 0.15 (− 0.44 to 0.14)	0.313	− 0.03 (− 0.20 to 0.13)	0.686	1.000
Postgraduate year	0.07 (0.03 to 0.11)	< 0.001	0.01 (− 0.01 to 0.04)	0.352	1.000
Duration of training	0.34 (0.20 to 0.47)	< 0.001	0.07 (−0.06 to 0.21)	0.304	1.000
Skill repetitions	0.54 (0.50 to 0.57)	< 0.001	0.06 (0.01 to 0.12)	0.032	0.899
Perceived learning behavior	0.99 (0.95 to 1.04)	< 0.001	0.69 (0.61 to 0.78)	< 0.001	**< 0.001**
Base specialty		< 0.001		0.141	1.000
- Intensive care medicine	Ref.		Ref.		
- Anesthesiology	−0.28 (−0.62 to 0.07)	0.113	− 0.24 (− 0.47 to − 0.00)	0.050	1.000
- Internal medicine	− 0.80 (−1.10 to − 0.49)	< 0.001	−0.12 (− 0.38 to 0.13)	0.340	1.000
- Surgery	−0.35 (− 0.77 to 0.07)	0.102	0.09 (− 0.24 to 0.41)	0.609	1.000
- Others	−0.62 (−1.21 to − 0.03)	0.039	0.03 (− 0.43 to 0.49)	0.903	1.000
Skill type		< 0.001		< 0.001	**< 0.001**
- Chest drain insertion	Ref.		Ref.		
- Tracheotomy	−0.78 (−1.01 to − 0.56)	< 0.001	− 0.40 (− 0.57 to − 0.22)	< 0.001	**< 0.001**
- Cricothyroidotomy	− 1.13 (− 1.36 to − 0.91)	< 0.001	− 0.45 (− 0.64 to − 0.27)	< 0.001	**< 0.001**
- Pericardiocentesis	− 1.27 (− 1.49 to − 1.05)	< 0.001	− 0.48 (− 0.67 to − 0.30)	< 0.001	**< 0.001**
- ECMO cannula insertion	−1.23 (− 1.46 to − 1.01)	< 0.001	− 0.52 (− 0.70 to − 0.33)	< 0.001	**< 0.001**
- Endotracheal intubation	0.14 (− 0.08 to 0.36)	0.220	− 0.06 (− 0.23 to 0.12)	0.512	1.000
- Central line insertion	0.82 (0.59 to 1.04)	< 0.001	0.12 (− 0.09 to 0.32)	0.257	1.000
- Arterial line insertion	1.02 (0.80 to 1.24)	< 0.001	0.13 (− 0.08 to 0.35)	0.223	1.000

The standard deviation of variance components of the multivariable model was 0.0, 0.24, and 0.47, respectively for hospitals, participants, and residuals

### Expected supervision level

Multivariable analysis indicated associations between supervision level and perceived learning behavior (*p* < 0.0001), skill repetitions (*p* < 0.0001), and skill type (tracheotomy, cricothyroidotomy, pericardiocentesis, and ECMO cannula insertion, all *p* < 0.001) (Table 7).

**Table 7:** Univariable models and multivariable models for dependencies of expected supervision level

Variable	Univariable models Crude coef. (95% CI)	***p***-value	Multivariable models Adjusted coef. (95% CI)	***p***-value	Corrected ***p***-value
Gender (female)	−0.24 (− 0.51 to 0.02)	0.071	− 0.19 (− 0.33 to − 0.04)	0.012	0.354
Postgraduate year	0.10 (0.07 to 0.13)	< 0.001	0.03 (0.00 to 0.05)	0.026	0.762
Duration of training	0.37 (0.25 to 0.48)	< 0.001	0.05 (−0.06 to 0.17)	0.371	1.000
Skill repetitions	0.57 (0.54 to 0.61)	< 0.001	0.12 (0.07 to 0.17)	< 0.001	**< 0.001**
Perceived learning behavior	1.02 (0.98 to 1.06)	< 0.001	0.65 (0.57 to 0.72)	< 0.001	**< 0.001**
Base specialty		< 0.001		0.850	1.000
- Intensive care medicine	Ref.		Ref.		
- Anesthesiology	−0.08 (−0.38 to 0.23)	0.621	− 0.01 (− 0.21 to 0.19)	0.945	1.000
- Internal medicine	−0.71 (− 0.99 to − 0.43)	< 0.001	0.07 (− 0.15 to 0.28)	0.541	1.000
- Surgery	− 0.43 (− 0.81 to − 0.06)	0.023	0.03 (− 0.25 to 0.31)	0.838	1.000
- Others	− 0.62 (−1.15 to − 0.10)	0.020	−0.10 (− 0.49 to 0.28)	0.595	1.000
Skill type		< 0.001		< 0.001	**< 0.001**
- Chest drain insertion	Ref.		Ref.		
- Tracheotomy	−0.77 (−0.98 to − 0.56)	< 0.001	− 0.35 (− 0.50 to − 0.20)	< 0.001	**< 0.001**
- cricothyroidotomy	−1.09 (− 1.30 to − 0.88)	< 0.001	− 0.37 (− 0.53 to − 0.21)	< 0.001	**< 0.001**
- pericardiocentesis	−1.20 (− 1.41 to − 0.99)	< 0.001	−0.38 (− 0.54 to − 0.22)	< 0.001	**< 0.001**
- ECMO cannula insertion	− 1.23 (− 1.44 to − 1.02)	< 0.001	− 0.48 (− 0.64 to − 0.32)	< 0.001	**< 0.001**
- Endotracheal intubation	0.10 (− 0.11 to 0.31)	0.341	− 0.12 (− 0.27 to 0.03)	0.123	1.000
- Central line insertion	0.90 (0.69 to 1.11)	< 0.001	0.14 (− 0.03 to 0.31)	0.107	1.000
- Arterial line insertion	1.20 (0.99 to 1.41)	< 0.001	0.24 (0.05 to 0.42)	0.011	0.349

The standard deviation of variance components of the multivariable model was 0.06, 0.2, and 0.4, respectively for hospitals, participants, and residuals

## Discussion

Current educational concepts neglect the association between residents’ learning behavior and the faculty teaching style and/or supervision level. Differences between novice, beginner, and proficient are recognized; however, achieving progress from one stage to the next and/or how to adapt supervision remains unclear [19, 20, 22].

Based on Hersey’s Situational Leadership Theory, residents’ learning behavior may be determined at least in part by their experience [9]. A given resident’s learning behavior may require a distinct teaching style and supervision level for appropriate learner-centered education.

In this cross-sectional multicenter study, a low number of skill repetitions appeared to be related to lower readiness (a combination of ability and willingness) to perform skills, which led to lower rating of perceived learning behavior (Table 1), and supervision level. The study may illustrate that residents’ expectation in terms of teaching behavior and supervision change as a function of their perceived learning behavior.

The Cognitive Load Theory by Sweller et al. assumes that the cognitive load varies directly with the “mental effort” and inversely with the available cognitive capacity [23, 24]. Therefore, skills performed with a low number of repetitions caused a greater cognitive load [25]. In less experienced residents the greater cognitive load and the required mental effort may influence their ability and/or willingness of residents and may lead to a distinct learning behavior (Novice or Advanced Beginner, Table 1). On the other hand, more experienced residents may have a lower cognitive load and exerts less mental effort when performing a skill. This may influence their ability and/or willingness, with a tendency to show different learning behavior, such as being proactive, autonomous, and transparent about their performance (Competent or Proficient, Table 1). Additionally, residents gaining experience in given skills typically gain in competence (reducing their cognitive load), which may change their learning behavior.

Less complex skills (e.g., arterial line insertion, central venous line insertion) may needs less mental effort than complex skills (e.g., ECMO cannula insertion, pericardiocentesis). Hence, it appeared that perceived learning behavior, expected teaching style, and expected supervision level may depend on skill repetitions, and skill type, as well as on skill complexity [23, 24].

Interestingly, it seems that internal medicine residents undergoing ICU training may perceive their learning behavior with regard to technical ICU skills differently than residents with intensive care medicine as base specialty. This may be because the skills and/or techniques concerned are infrequently encountered by internal medicine residents during their pre-ICU educational curriculum. Therefore, uncertainty and limited confidence may lead to limited willingness followed by limited readiness, which may affect learning behavior (Fig. 1) [14, 26].

Overall, appeared that faculty’s choice of teaching style and supervision level should be driven by residents’ learning behavior, thus ensuring the residents’ capacity to learn and perform effectively [14]. However, it should be noted that there appears to be a continuum of teaching styles and supervision levels rather than separate steps.

Residents’ self-assessment of learning behavior may lead to a specific expected teaching style or supervision level [27]. Self-assessment has several limitations and may not reflect residents’ objective performance [28, 29]. However, residents’ and faculty’s’ estimations of the number of skill repetitions needed in order to achieve competency appear similar [30]. Despite this, some residents who had no or very few skill repetitions expected minimal supervision (Table 4). However, it appeared that in addition to the numbers of skill repetitions, the skill type may also influence the supervision level. To clarify the dependence between numbers of skill repetitions and skill type or skill difficulty, further investigations are recommended. Moreover, it should be investigated whether and when residents are ready to perform skills unsupervised. Furthermore, it may be helpful to estimate the resident’s needs, recognizing that the best learning performance occurs when learning tasks force residents to leave their “comfort zone” [31]. It should be noted that residents’ concerns for patient safety, fears of social evaluation, or repercussions on evaluations might affect their confidence, commitment, or motivation and therefore their subsequent learning behavior (Fig. 1) and therefore the expected teaching style and supervision level.

### Implications for learner-centered education

Residents’ learning behavior appeared to be affected by their readiness (i.e., ability and willingness combined) to perform a distinct skill (Fig. 1) [14]. Readiness, in turn, may be influenced by the number of skill repetitions previously carried out [24, 32]. Hence, the level of learning behavior may increase, and it appeared that teaching styles and supervision levels should be selected independently of the duration of training and postgraduate year. This is in line with the concept of CBME, which emphasizes the importance of acquired competencies rather than the amount of time spent in training [33]. The numbers of skill repetitions may be necessary so that the resident can acquire experience and actively gain knowledge. With regard to the constructivism learning theory, stepwise adaptation of teaching styles and supervision levels may help the residents to construct new knowledge based on previous personal knowledge and experience [34, 35]. However, the appropriate time to let residents perform a specific procedural skill unsupervised remains unclear.

In addition, the individual adaptation of the teaching style and supervision level to the learning of individual skills corresponds to the learner-centeredness embodied in CBME [36]. Hence, the faculty may wish to take into account the residents’ learning behavior, driven mainly by skill complexity and the number of skill repetitions, to deliver the appropriate teaching style and supervision level. Here, faculty development programs may provide theoretical concepts, exercises, and simulations regarding the Situational Leadership Theory in the educational setting [37, 38].

### Limitations

This study has several important limitations. First, the small size of the sample means that the results should be interpreted with caution. Further investigations with a larger sample sizes should be carried out to verify our findings. Furthermore, the study design meant that only self-assessed data were discussed, with all inherent limitations [39]. Hence, the validity of self-assessed learning behavior should be clarified in further investigations examining the underlying components of the residents’ ability (knowledge, experience, and manual dexterity) and willingness (confidence, commitment, and motivation; Fig. 1). Furthermore, investigations should focus on faculty’s assessment of residents’ learning behavior and the implications for teaching style and supervision level. Here, future study designs should use summative assessments to clarify the relationship of teaching styles and supervision levels with numbers of skill repetitions. Also, other settings (e.g., surgery, internal medicine) should be investigated with regard to application of the Situational Leadership Theory. In this study, we focused on procedural skills. However, further investigations are needed to explore the current research question in other skill types (e.g., diagnostic skills, non-technical-skills). Importantly, we report associations rather than causal relationships.

## Conclusion

For effective learner-centered education, it appears useful to recognize that residents’ learning behavior is affected by the number of skill repetitions and skill complexity. Therefore, the faculty may wish to take into account residents’ learning behavior in order to deliver appropriate teaching styles and supervision level.

## Supplementary Information

**Additional file 1.**

## Data Availability

All data generated or analyzed during this study are included in this publication.

## Abbreviations

ICU: Intensive care unit
PGY: Postgraduate year
ECMO: Extracorporeal membrane oxygenation
CBME: Competency-based medical education
EPA: Entrustable professional activities
SD: Standard deviation
CI: Confidence interval
Coef.: Coefficient
