# Supplementary material for: Learner-centered education: ICU residents’ expectations of teaching style and supervision level
Source: BMC Med Educ. 2021 Jul 31;21:411. doi: 10.1186/s12909-021-02844-z (PMC8325219; doi:10.1186/s12909-021-02844-z)
Supplement: Supplementary file 1 — Additional file 1. [file 12909_2021_2844_MOESM1_ESM.docx]

**Learner-centered education: ICU resident’s expectations on teaching style and supervision level**

Bjoern Zante MD MME^1^, Jennifer Klasen MD MME^2^

1. Department of Intensive Care Medicine, Inselspital, Bern University Hospital, University of Bern, Bern, Switzerland
2. Clarunis, Department of Visceral Surgery, University Centre for Gastrointestinal and Liver Diseases, University Hospital Basel, Basel, Switzerland Jennifer.Klasen@clarunis.ch

**Supplemental**
